# Protracted dormancy of pre-leukemic stem cells

**DOI:** 10.1038/leu.2015.132

**Published:** 2015-07-14

**Authors:** A M Ford, M B Mansur, C L Furness, F W van Delft, J Okamura, T Suzuki, H Kobayashi, Y Kaneko, M Greaves

**Affiliations:** 1 Centre for Evolution and Cancer, The Institute of Cancer Research, London, Sutton, UK; 2 Paediatric Haematology-Oncology Program, Research Centre, Instituto Nacional de Câncer, Rio de Janeiro, Brazil; 3 Newcastle Cancer Centre, NICR, Newcastle, UK; 4 Department of Pediatrics, National Kyushu Cancer Centre, Minami-ku, Japan; 5 Department of Hematology, Saitama Cancer Centre, Ina, Japan

## Abstract

Cancer stem cells can escape therapeutic killing by adopting a quiescent or dormant state. The reversibility of this condition provides the potential for later recurrence or relapse, potentially many years later. We describe the genomics of a rare case of childhood *BCR-ABL1*-positive, B-cell precursor acute lymphoblastic leukemia that relapsed, with an acute myeloblastic leukemia immunophenotype, 22 years after the initial diagnosis, sustained remission and presumed cure. The primary and relapsed leukemias shared the identical *BCR-ABL1* fusion genomic sequence and two identical immunoglobulin gene rearrangements, indicating that the relapse was a derivative of the founding clone. All other mutational changes (single-nucleotide variant and copy number alterations) were distinct in diagnostic or relapse samples. These data provide unambiguous evidence that leukemia-propagating cells, most probably pre-leukemic stem cells, can remain covert and silent but potentially reactivatable for more than two decades.

## Introduction

Recurrence or relapse of cancer many years^1, 2, 3^ or occasionally decades^4^ after an initial diagnosis has been frequently recorded. These observations raise difficult issues related to presumptions of cure, risk assessment and monitoring of residual disease. A plausible mechanism for persistent, covert cancer cells during and after treatment is provided by the observation that some cancer stem cells can adapt a reversible quiescent or dormant state in which they are relatively resistant to radiation and chemotherapy.^5, 6, 7, 8^ However, the assumption is usually made that late recurring cancer is a derivative of the original clone at diagnosis, evidence for which is very limited, with the exception of some acute leukemias where physiological rearrangement of immunoglobulin genes (*IGH/IGK*) provide clone-specific markers.^9, 10, 11^

## Materials and methods

### Targeted capture libraries, cloning and sequencing of gene fusions

A cell line, MR-87, was established from the original leukemic cells of the 4-year-old patient and it showed the same immunophenotype and karyotype of the diagnostic leukemic cells. These cells were also shown to express the p190 BCR-ABL1 protein.^12^ Illumina paired-end libraries (Illumina, San Diego, CA, USA) covering the entire genomic regions of the *BCR*, *ABL1* and *IKZF1* genes were prepared from the MR87 cell line DNA (diagnosis) using the Agilent SureSelectXT 2 Custom (1–499 kb) DNA bait library (Agilent Technologies, Santa Clara, CA, USA). The custom libraries were sequenced on a HiSeq2500 (Illumina) to a coverage depth of 99 ×. Casava software (v1.8, Illumina) was used to make base calls and demultiplex the sequencing data and the genomic fusion breakpoints of *BCR-ABL1* and *IKZF1* were roughly determined using Burrows–Wheeler Aligner and Breakdancer software (The Genome Institute, St Louis, MO, USA). The *BCR-ABL1* breakpoint fusion was predicted based on the location of read pairs that mapped to the fusion partners and the average fragment size of the capture library (320 bp). Using GRCh37.p13, the predicted breakpoint region in *BCR* was at chr22:23533568–23533950 (intron 1) and the breakpoint region in *ABL1* was expected at chr9:133608500–133608811 (intron 1). A large deletion in *IKZF1* (~50 kb) was observed between regions chr7:50412887–50463541. PCR primers were then designed to span the putative breakpoints using Primer3 plus (www.primer3plus.com/). Primers used for cloning the *BCR-ABL1* fusion were: 5′-GTCAAAGCATTTTCCCCTGC-3′ and 5′-TCTTGATACTGGGTTGGCTGC-3′, and for the *IKZF1* deletion were: 5′-GTCCTGGGTTTAAGCTTCAGTTCTCTGCCT-3′ and 5′-GGGTTGATAAGGAGGGTTTTGTGTCCCAGT-3′. Patient-specific gene fusions were amplified using AccuPrime *Taq* DNA Polymerase High Fidelity (Life Technologies, Carlsbad, CA, USA) and PCR products sequenced using BigDye Terminator v1.1 and an ABI-3730xl Genetic Analyzer (Applied Biosystems, Warrington, UK). Sequences were aligned by BLAST (http://blast.ncbi.nlm.nih.gov/Blast.cgi).

### Screening for *IG* and *TCR* gene rearrangements

DNA was extracted from diagnostic (MR87) and relapse cells (peripheral blood and bone marrow). PCR amplification of immunoglobulin (*IG*) heavy-chain variable–diversity–joining (*IGH* V(D)J; complete and incomplete), *IG*κ variable–joining (*IGK-*VJ), *IGK* Vκ-deleting element (*Kde*), intron recombination signal sequence–*Kde*, *IG*λ (*IGL*), T-cell receptor-β (*TCRB*), *TCR*γ (*TCRG*) and *TCR*δ gene rearrangements were performed using primers and conditions recommended by the BIOMED-2 Consortium.^13^ The 6-carboxyfluorescein labelled products were analysed using an ABI 3500 Genetic Analyzer (Applied Biosystems), clonality was assessed by GeneScan software (Applied Biosystems) and the results were interpreted in accordance with the EuroClonality/BIOMED-2 guidelines.^14^ PCR products were cloned into pCR2.1 (Life Technologies), sequenced and analysed as above. Junction analyses were performed using IgBLAST (www.ncbi.nlm.nih.gov/igblast/) and the ImMunoGeneTics database (www.imgt.org/).

### Genome-wide copy number analysis

Single-nucleotide polymorphism array analysis was carried out using Affymetrix SNP 6.0 arrays (Affymetrix, Santa Clara, CA, USA), according to the manufacturer's instructions, on DNA extracted using standard methods from the diagnostic cell line (MR87) and from relapse bone marrow. Genotyping and generation of quality control data were performed in Genotyping Console v4.1.4 software (Affymetrix). The sample files were scrutinised for copy number alteration (CNAs) by visual inspection and by using the Partek Genomics Suite 6.6 software (Partek, St Louis, MO, USA). Copy number was determined by normalisation to Partek-distributed baseline files, which comprise 270 Hapmap files, using a genomic segmentation algorithm.

### Whole exome sequencing

Exome capture was performed using the Agilent SureSelect Human All Exon V5 kit as per the manufacturer's instructions (Agilent) and sequenced by Illumina paired-end sequencing (protocol v1.2). Briefly, DNA was sheared by fragmentation (Covaris, Woburn, MA, USA), purified using Agencourt AMPure XP beads (Beckman Coulter, Pasadena, CA, USA) and the resulting fragments analysed on an Agilent 2100 Bioanalyzer. Fragment ends were repaired and adaptors ligated to the fragments, and the library was purified using beads. After amplification and hybridisation with biotinylated RNA baits, bound genomic DNA was purified with streptavidin-coated magnetic Dynabeads (Life Technologies) and re-amplified to include barcoding tags before finally pooling for sequencing on an Illumina HiSeq 2000.

Exome analysis was completed in Oxford Gene Technology's (Begbroke, UK) exome pipeline. Briefly, reads were aligned to the human genome reference sequence GRCh37 using Burrows–Wheeler Aligner 0.6.2.^15^ Local realignment was performed around indels with the Genome Analysis Toolkit (GATK v1.6) IndelRealigner.^16^ Optical and PCR duplicates were marked in BAM files using Picard 1.107 (http://picard.sourceforge.net). Original HiSeq base quality scores were recalibrated using GATK TableRecalibration and per-sample variants called with GATK UnifiedGenotyper (Broad Institute, Cambridge, MA, USA). Indels and single-nucleotide variants (SNVs) were hard filtered according to the Broad Institute best-practice guidelines, to eliminate false-positive cells.

Copy number variants, somatic SNV and somatic indels were identified between presentation and relapse samples using VarScan2.^17^ Variant annotation was performed with a modified version of Ensembl Variant Effect Predictor.^18^

## Results

### Clinical and haematological features of case

A brief case report of the patient was already published^19^ and is summarised as follows. A 4-year-old boy was diagnosed as having precursor B-cell acute lymphoblastic leukemia (ALL) but with a mixed lympho-myeloid phenotype: positive for myeloperoxidase, CD13^+^, CD10^+^ and CD19^+^. Cytogenetics on leukemic cells showed 46,XY,9p−,t(9q+22q−), indicating Ph^+^ pre-B-ALL. Reverse transcriptase-PCR confirmed the presence of the minor breakpoint (p190) *BCR-ABL1* fusion.

The patient was treated with chemotherapy and achieved complete remission. Eight weeks after the diagnosis, he developed a central nervous system relapse, which was successfully treated with cranial irradiation and intrathecal drug administration. Three months after the diagnosis, he received a bone marrow transplant (BMT) from his human leukocyte antigen-identical (non-twin) brother when in the second complete remission. BMT transplantation was successful and no major complications were observed.

At the age of 25 years (20 years after BMT transplantation), the patient presented with general fatigue. His white blood cell count was 16.7 × 10^9^/l with 7% blasts and his bone marrow aspirates showed leukemic cells with a myeloid immunophenotype positive for CD13 and CD33, and negative for CD10 and CD19. The leukemic cell karyotype was 46,XY,t(9;22)(q34;q11) × 2 plus other complex abnormalities. He was tentatively diagnosed as having a relapse of the initial Ph^+^ pre-B-ALL and received intensive chemotherapy resulting in complete remission. He underwent the second BMT transplant from a human leukocyte antigen-identical unrelated donor but had bone marrow relapse 35 weeks after the second BMT transplant and subsequently died of the disease.

### Diagnostic and late relapse clones share an identical *BCR-ABL1* fusion sequence

**Figure 1:**
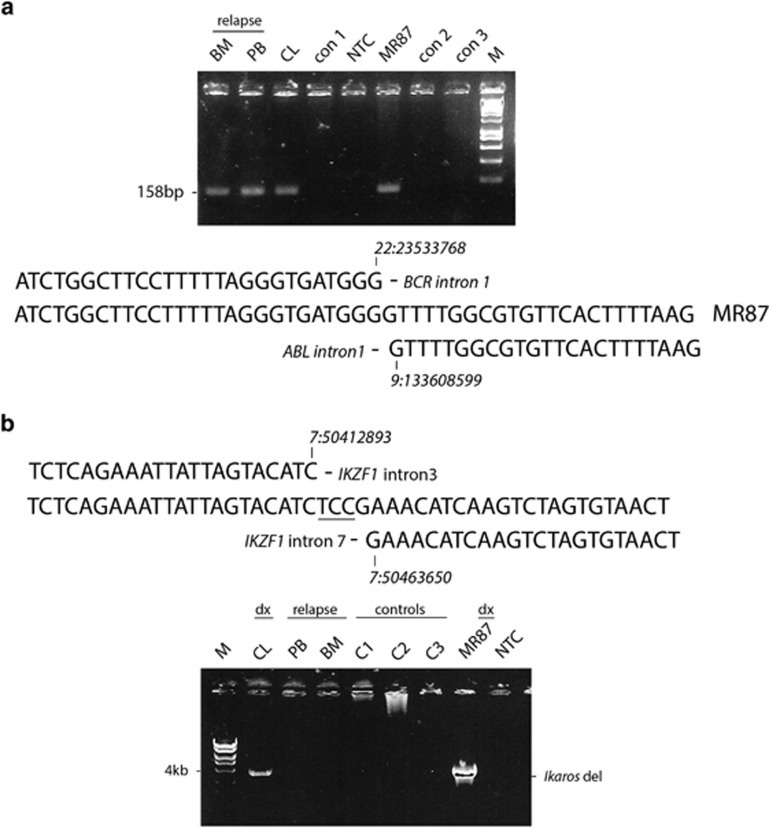
Diagnostic and late relapse clones share an identical *BCR-ABL1* fusion sequence with a discordant intra-gene deletion of *IKZF1*. (**a**) Upper panel: PCR primers that span the *BCR-ABL1* breakpoint identified in diagnostic material (MR87, CL) were used to interrogate DNA isolated at relapse (bone marrow (BM) and peripheral blood (PB)). An identical product is seen in all patient samples but not in leukemia controls. Lower panel: DNA sequence of the *BCR-ABL1* fusion and comparison with wild-type *BCR* and *ABL1* gene sequences (GRCh37.p13). The DNA sequence was identical in both diagnostic and relapse samples. (**b**) Upper panel: DNA fusion sequence (GRCh37.p13) of the *IKZF1* deletion at diagnosis reveals a 50-kb deletion between introns 2 and 7. Lower panel: the deletion/fusion product present at diagnosis (MR87 and CL) is not observed at relapse (BM and PB) or in DNA from leukemia controls.

The putative breakpoint regions of the *BCR* and *ABL1* genes were identified by targeted whole-genome sequencing of DNA isolated from the cell line (MR87) derived from patient cells at diagnosis. PCR primers were designed 5′ to the putative breakpoint in *BCR* and 3′ to that in *ABL1*, and the patient-specific *BCR-ABL1* gene fusion was amplified, cloned and sequenced. The breakpoint detected in the *BCR* gene occurred within intron 1 at GRCh37.p13 position ch22:23533768 and within intron 1 of the *ABL1* gene at position ch9:133608599 (Figure 1). The breaks in both *BCR* and *ABL1* are therefore outside the recognised cluster regions described for Ph+ leukemia.^20^ The same set of PCR primers were next used to interrogate DNA from the peripheral blood and bone marrow at relapse and an identical sized fusion product was obtained. Cloning and sequencing of the relapse fusion products proved the *BCR-ABL1* fusion sequence to be identical to that present at diagnosis (Figure 1a).

### Genome-wide copy number analysis

SNP 6.0 analysis on DNA isolated from the diagnostic cell line showed the following recurrent leukemia CNA: deletion of *MTAP*, *CDKN2A*/*B*, *PAX5*, 6q14.1–6q16.1 and *IKZF1*. In addition, amplification of *MDM2* was noted. Relapse material was discordant for the diagnostic CNA drivers (Table 1); however, copy number loss of 9p was demonstrated (including loss of the same genes deleted at diagnosis: *CDKN2A/B*, *MTAP* and *PAX5*) but results clearly demonstrated that this 9p deletion was a re-iterative event with distinct breakpoints to the diagnostic sample (Supplementary Tables S1 and S2, and Supplementary Figures S1A–F). Potential drivers newly acquired in the relapse material also included deletion of the majority of chromosome 21q, gain of chromosome 20 and deletion of 8p (Supplementary Table S2).

Given that deletions in the tumour suppressor gene *IKZF1* are considered a driving force of leukemogenesis, we used targeted sequencing of diagnostic DNA to design PCR primers that spanned the putative boundaries of the 50 kb *IKZF1* intra-gene deletion. Subsequent PCR produced an ~4 kb amplification product that was further cloned and sequenced (Figure 1b). The 5′-breakpoint was determined at GRCh37.p13 position 7:50412893 and the 3′-breakpoint at position 7:50463650, with loss of 50 757 bp of DNA and the random insertion of 3 nucleotides (Figure 1b). Using the same primer set, we could not detect a deletion in the *IKZF1* gene by conventional PCR or sensitive quantitative PCR in the peripheral blood or bone marrow at relapse (Figure 1b and Supplementary Figures S2 and S5). These data indicate that some genes (*IKZF1* and *CDKN2A*) were subject to reiterated CNA in diagnosis and relapse but no CNA was preserved from diagnosis to relapse.

### Clonality of immunoglobulin gene rearrangements at diagnosis and relapse

**Figure 2:**
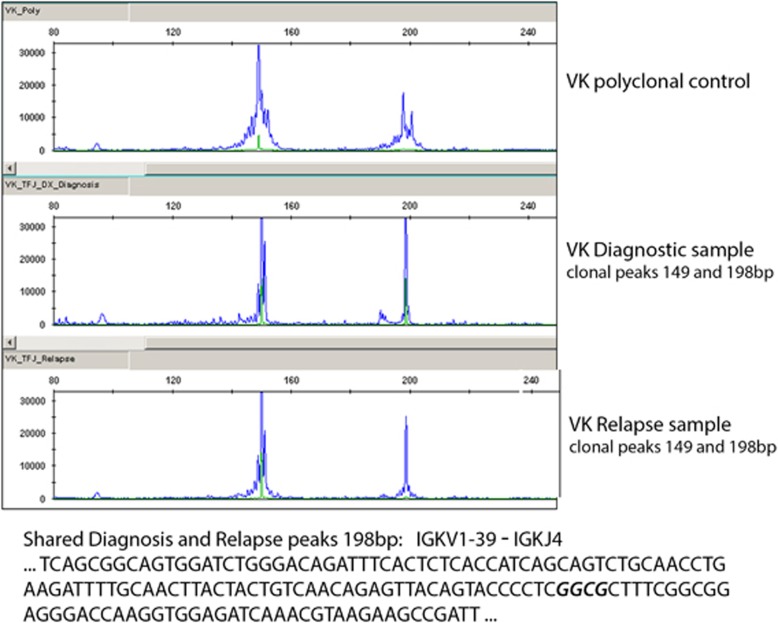
Clonality of immunoglobulin light chain gene rearrangements at diagnosis and relapse. Upper panel: PCR amplifications of *IGλ* (*IGL* V(N)J) rearrangements at diagnosis and relapse were assessed by GeneScan software. Lower panel: DNA sequence analysis of the major V(N)J rearrangement identified at diagnosis shows an identical sequence to that observed at relapse. N region insertion is shown in bold italics.

Screening for clonal *IG* and *TCR* gene rearrangements to assess clonality was performed on both the diagnostic and relapse DNA using multiplex PCR reactions and ABI GeneScan profiling. Clonal rearrangements were identified in both *IGH* VDJ (FR1 and 2) and *IGL* VJK reactions (Figure 2 and Supplementary Figure S3) with weaker clonal rearrangements observed in *TCRBB/C* and in *TRG1* (data not shown). A V(N)JK light-chain rearrangement was shown to be identical between diagnosis and relapse (Figure 2), and the two major *IGH* V(N)D(N)J peaks identified at 294 and 335 bp at diagnosis were similarly shown to have identical sequences to the respective minor peaks observed at relapse (Supplementary Figure S3). However, the two major peaks identified in relapse at 330 and 341 bp were not detected in diagnostic material by conventional or quantitative PCR (Supplementary Figure S4). One interpretation of these data is that the ‘founder' *IGH* rearrangement present in the diagnostic samples underwent further rearrangement in relapse. Taken together, these data further suggest that the diagnostic and relapse clones may have arisen from a pre-leukemic progenitor cell already partially committed to the B-cell lineage. However, the myeloid or acute myeloblastic leukemia immunophenotype seen in relapse indicates that the leukemia was essentially ‘mixed lympho-myeloid' and may have arisen in a cell with some myeloid differentiation capacity despite the clonal *IGH* rearrangements present in the bulk cell progeny.

### Whole exome sequencing analysis

We performed whole exome sequencing on patient DNAs isolated at diagnosis, remission (germline) and relapse. All possible cross-comparisons between these three time points were assessed in the data analyses. In terms of somatic alterations, at diagnosis we identified, before filtering, a total of 2189 SNVs and 648 insertions and/or deletions. At relapse we identified 7320 SNVs and 1567 indels.

In further analyses, we highlighted relevant functional alterations. In the diagnostic sample, after filtering the data by read depth (between 30–170 ×), coding areas only and SNVs predicted to alter protein structure and deleterious/possibly damaging at the protein level (Ensembl Variant Effect Predictor), we detected 92 somatic SNVs and 59 indels. We selected those genes with functions known to be associated with cancer of which there were 12—SNVs: *NOTCH2*, *PIK3CG*, *IL2RB*, *BAI3*, *FREM2* and *RERE*; indels: *UTRN*, *CDHR3*, *NCOA5*, *CABYR*, *HOTAIR* and *FOLH1* (Table 2). In the relapse sample after a similar filtration, we identified 156 SNVs and 46 indels, and identified 10 potential ‘driver' genes—SNVs: *THOC6*, *VANGL2*, *THBS1*, *STAT2* and *ACY1*; indels: *NBEAL1*, *SMG7*, *TRIM29*, *FANCG* and *FAM186A* (Table 2). All 22 genes have previously been shown to have a relevant role in tumourigenesis or have potential to be a ‘driver' of leukemogenesis. We confirmed selected heterozygous point mutations or indels by Sanger sequencing, that is, *NOTCH2*, *HOTAIR*, *STAT2* and *FANCG* (data not shown). None were shared between the diagnostic and relapse samples, and were absent in remission (control, constitutive DNA).

## Discussion

**Figure 3:**
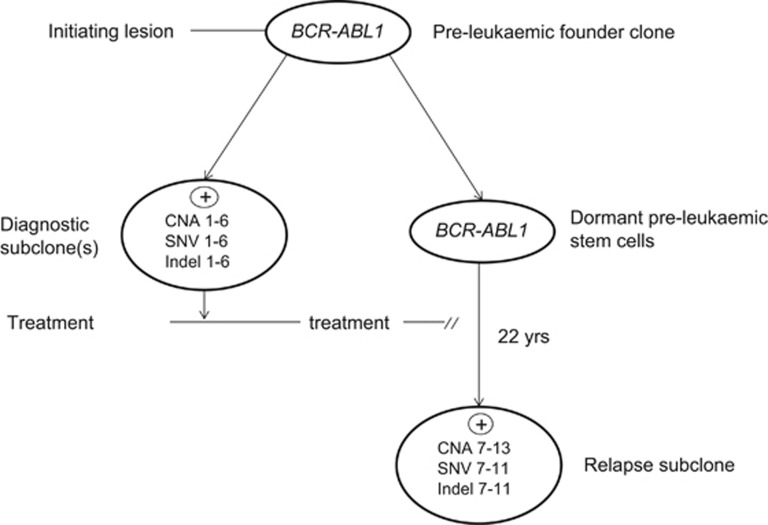
Model for the pre-leukemic origins of very late relapse. CNA, SNV and indel numbers—distinctive mutations found in diagnostic and relapse clones (numbers 1–13 refer to aberrations noted in Tables 1 and 2).

The identity of shared and clone-specific genotypic sequences in this patient's diagnostic and very late relapse leukemia cell population provides unambiguous evidence that the relapse derived, after 22 years, from descendent progeny of the original founder clone (Figure 3). Late relapses derived from the founder diagnostic clones in ALL have been described before,^9, 10^ but this is the longest dormancy interval recorded with the possible exception of a case relapsing after 34 years in which the genetic evidence was very limited.^21^ It is striking that although the *BCR-ABL1* fusion gene was identical in the paired diagnostic/relapse samples, all other genetic abnormalities detected by the single-nucleotide polymorphism arrays as CNA or by exomic sequencing as SNVs were distinctive, although the same gene was in some reiteratively mutated (for example, *CDKN2A* and *IKZF1*). Reiterative CNA have been reported before in ALL^22, 23^ and the predominant mutational mechanism for these structural changes appears to be driven by the lymphoid recombinases RAG1/2.^24^ SNV in ALL have a different mutational mechanism involving APOBECs.^24^ It is unclear whether the predominance of CNA as recurrent changes in ALL is a reflection of the relative activity of these different mutational mechanisms, the prevalence of different selective pressures or differential functional impacts of CNA versus SNV on cellular fitness.

ALLs have multiple, genetically distinct stem cells at diagnosis.^22^ Our interpretation of the genomic data on this patient is that the long term surviving stem cells that spawned very late relapse derived from stem cells of a minor clone at diagnosis and most likely from a pre-leukemic clone that harboured a founder *BCR-ABL1* lesion but not other secondary genetic changes (Figure 3). Evidence for such pre-malignant clones in ALL with *BCR-ABL* or other founder lesions have been provided by comparative genetics of monozygotic twins with discordant ALL.^25, 26, 27^ Sharing of identical or clonotypic *BCR-ABL1* genomic fusions in monozygotic twins with concordant or discordant ALL but discordance of other genetic changes^27^ suggests that the *BCR-ABL1* fusion in such cases is an early or likely founder or initiation event spawning a pre-leukemic clone. Limited comparative genetics had previously suggested that some late relapses in ALL might be spawned by persistent pre-leukemic clones.^28, 29^ Immunophenotypically or genetically defined pre-leukemic cells have previously been shown to preferentially survive chemotherapy in ALL^30^ and acute myeloblastic leukemia.^31^ Recently, Zhang *et al.*^32^ reported a relapse after 17 years in a case of acute promyelocytic leukaemia. The comparative genetics in this case was also compatible with the relapse originating from pre-leukemic stem cells.

Many mechanisms have been proposed to explain protracted clinical dormancy of cancer including balanced proliferation, cell death, non-angiogenic phenotypes, negative signalling within stromal niches maintaining cells out of cycle and immune surveillance.^6, 7, 33^ Whatever the prevailing restraints, a late recurrence derived from the original clone requires that cells with self-renewal or stem cell potential survive to re-establish disease. In this respect, the recognised therapeutic resistance of quiescent cancer stem cells and residence in specialised bone marrow niches^33^ provides a basis for their survival in a dormant state, as we assume occurred in our patient.

Adopting dormancy as a survival strategy is not unique to cancer stem cells. Normal blood stem cells fluctuate between proliferative and quiescent or out of cycle phases.^34^ Bacteria ‘hunker down' or adopt a non-proliferative state when confronted with stressful conditions.^35^ The capacity of cancer stem cells to avoid lethal therapy by switching to a dormant state can be seen as a legacy of evolutionary programming of protective mechanisms for essential normal stem cells.

The case reported here reflects an extremely rare occurrence and does not conflict with the suggestion that cure in childhood ALL can be operationally defined by remission of 4 years post cessation of treatment when the risk of relapse is <1%.^10, 36^

Although many very late (>20 years) recurrences or relapses of cancer have been recorded, the assumption that this reflects re-awakening of the original clone or one of its subclones requires genetic scrutiny. Provided the diagnostic sample or biopsy is archived, this can be resolved, as in the current case, by comparative genomics.

## Figures and Tables

**Table 1:** Summary of ‘driver' CNAs present at diagnosis versus relapse detected by SNP analysis

	*Chr*	*Start*	*End*	*Cytoband*	*CNA loss/gain*	*Diagnosis*	*Relapse*	*Genes (max three listed)*
1	chr6	78095353	93349377	6q14.1–6q16.1	Loss	Present	Absent	*IRAK1BP1, PHIP, SYNCRIP*
2	chr7	50389071	50477011	7p12.2	Loss	Present	Absent	*IKZF1*
3	chr9	16523946	21901150	9p22.3–9p21.3	Loss	Present	Absent	*MLLT3, MTAP, FOCAD*
4	chr9	21901150	22347406	9p21.3	Homozygous Loss	Present	Absent	*CDKN2A, CDKN2B*
5	chr9	36412035	38493465	9p13.2–9p13.1	Loss	Present	Absent	*MELK, PAX5, FRMPD1*
6	chr12	68970862	69406845	12q15	Gain	Present	Absent	*RAP1B, NUP107, MDM2*
7	chr 7	50389060	50486601	7p12.2	Loss	Absent	Present	*IKZF1*
8	chr8	31254	43078119	8p23.3–8p11.21	Loss	Absent	Present	*ZNF596, FBXO25, DLGAP2*
9	chr9	6988784	21926959	9p24.1–9p21.3	Loss	Absent	Present	*MPDZ, MLLT3, MTAP*
10	chr9	21926959	22200408	9p21.3	Heterozygous Loss	Absent	Present	*CDKN2A, CDKN2B*
11	chr9	22200408	132070825	9p21.3–9q34.11	Loss	Absent	Present	*MOB3B, LINGO2, PAX5*
12	chr20	61305	62956154	20p13–20q13.33	Gain	Absent	Present	*DEFB125, DEFB126, TOP1*
13	chr21	15939316	48096958	21q11.2–21q22.3	Loss	Absent	Present	*CHODL,CXADR,NCAM2*

Abbreviations: CNA, copy number alteration; SNP, single-nucleotide polymorphism.

Based on NCBI37/hg19 assembly.

**Table 2:**
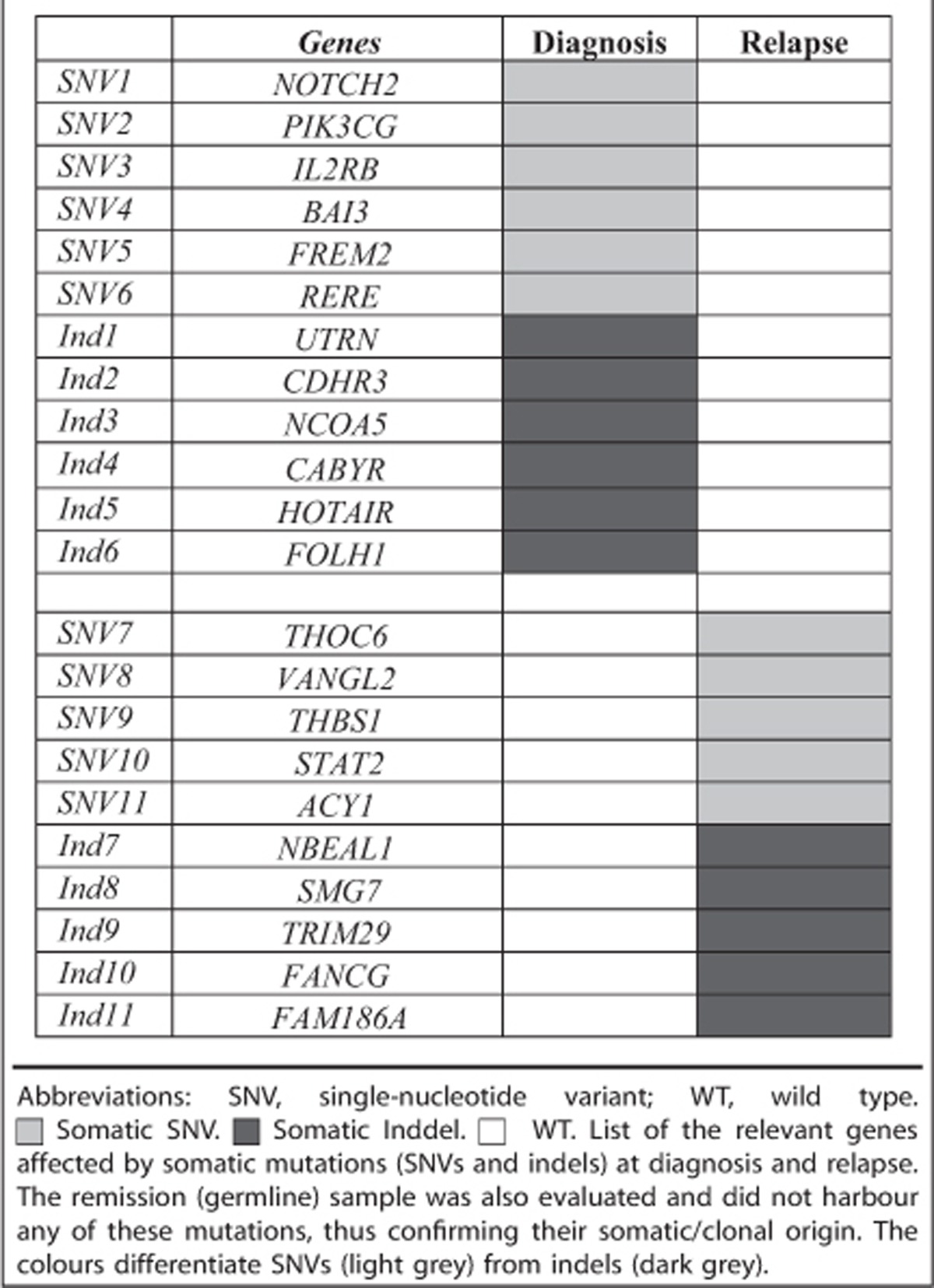
Principal mutations detected by whole exome sequencing of the patient DNA at diagnosis and relapse
