# Supplementary material for: Protracted dormancy of pre-leukemic stem cells
Source: Leukemia. 2015 Jul 14;29(11):2202–7. doi: 10.1038/leu.2015.132 (PMC4564945; doi:10.1038/leu.2015.132)

Ford et al Supplementary Information

Ford et al Supplementary Information

Ford et al Supplementary Information

**Figure S1. Discordant CNA;**  Figure S1A shows the pattern of CNA on chr 9 at diagnosis with deletion of 9p21.3 including *MTAP* plus loss of 2 copies of *CDKN2A*. *PAX5* is an additional separate CNA. Figure S1B shows a distinct 9p21.3 deletion at relapse. Figures S1C and S1D demonstrate the 6q loss and *MDM* amplification on chr 12q present at diagnosis but not at relapse. (Heatmaps are shown for diagnostic cell line (DIAG) and relapse bone marrow (Rel-BM). The copy number output displayed is for the diagnostic cell line.


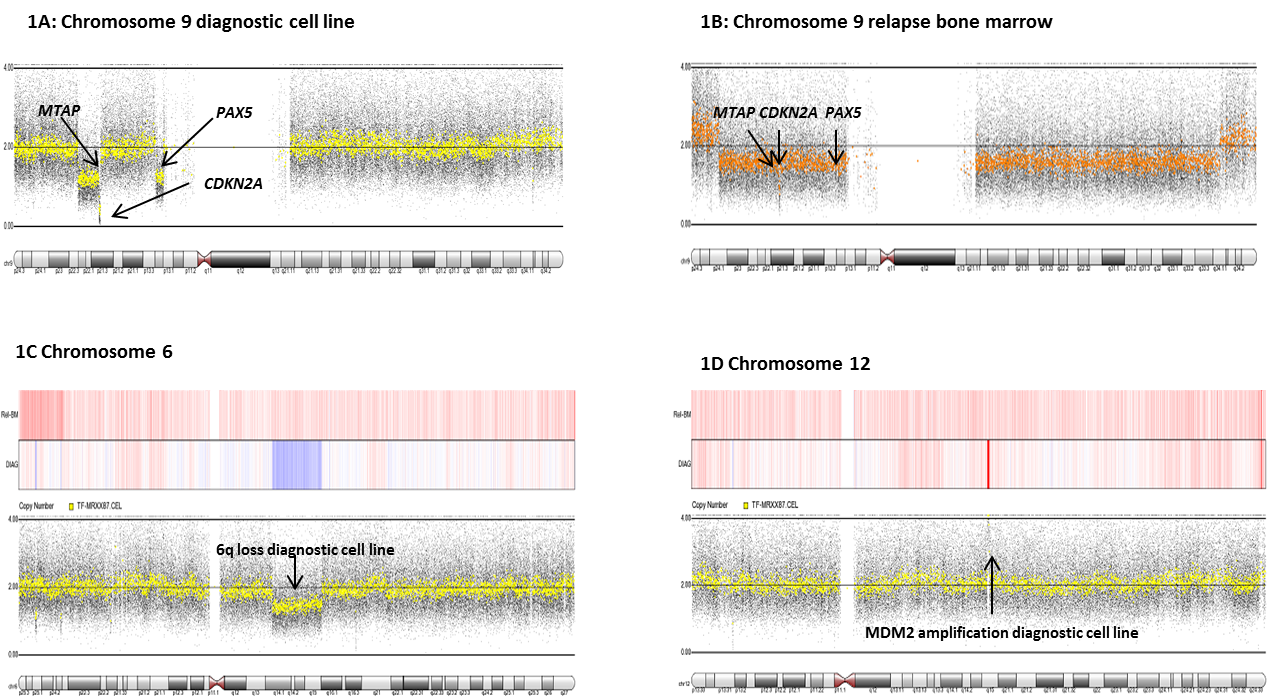


Ford et al Supplementary Information

**Figure S1E Diagnostic Cell line *IKZF1* deletion** (blue arrow)


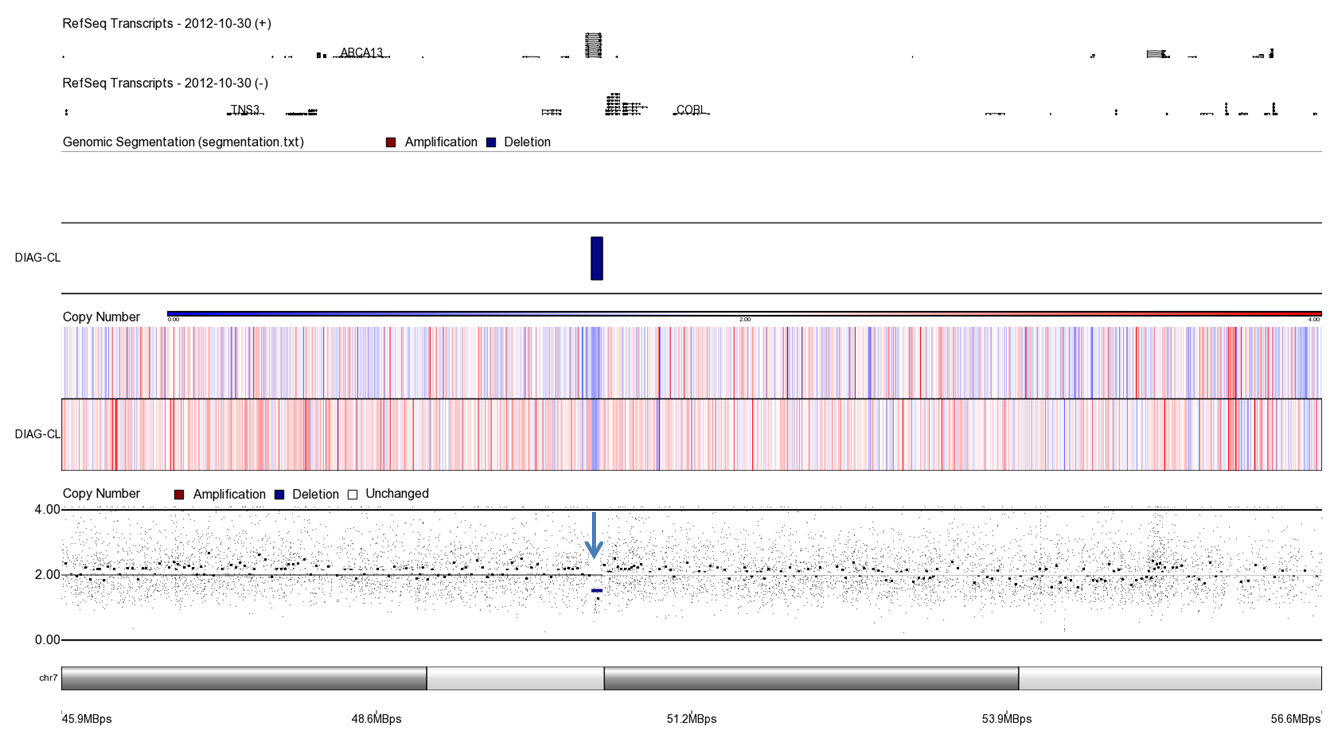


Ford et al Supplementary Information

**Figure S1F Relapse Bone marrow *IKZF1* deletion** (blue arrow)


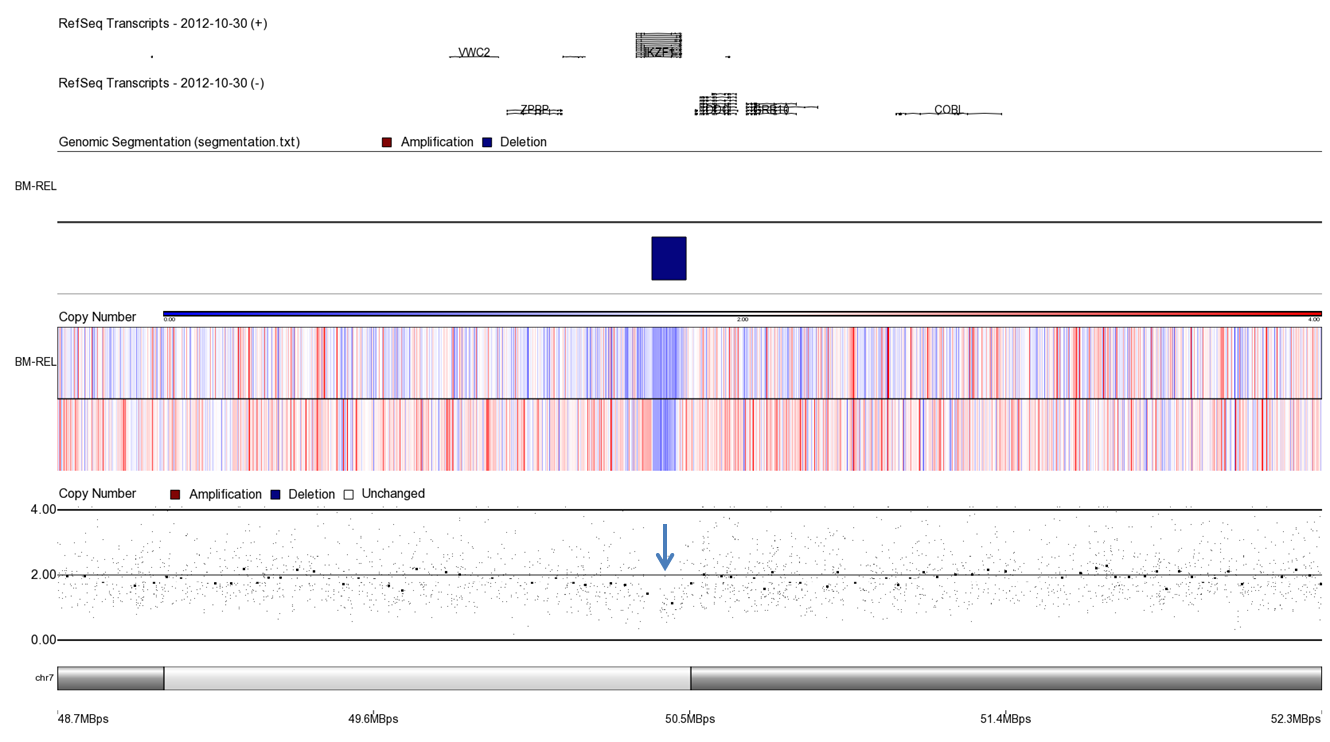


Ford et al Supplementary Information

**Figure S2 The *IKZF1* deletion observed at diagnosis is not detectable by Q-PCR at relapse**

Sequence of *IKZF1* deletion/fusion at diagnosis (see Figure 1) and Q-PCR primers used: *Forward, Probe, Reverse.*

Random insertions of N’s at the join are underlined.

AAATAATCTGAATTGACGGCATCCAGGGATCTCAGAAATTATTAGTACATCTCCGAAACATCAAGTCTAGTGTAACTGTTTCTTCTTCAAGGTGATTTG

Q-PCR plot from Bio-Rad CFX96.


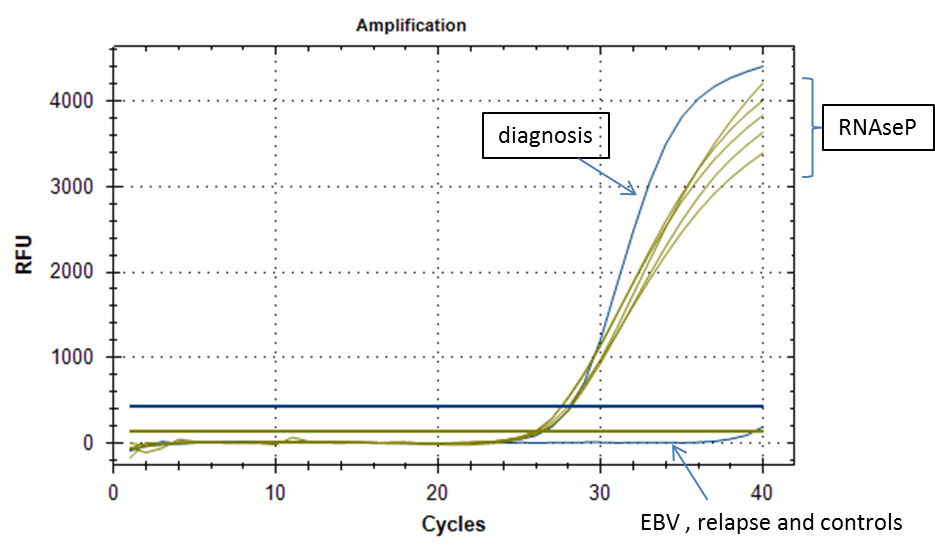


Ford et al Supplementary Information

**Figure S3 Immunoglobulin heavy chain gene clonality: diagnosis v relapse**


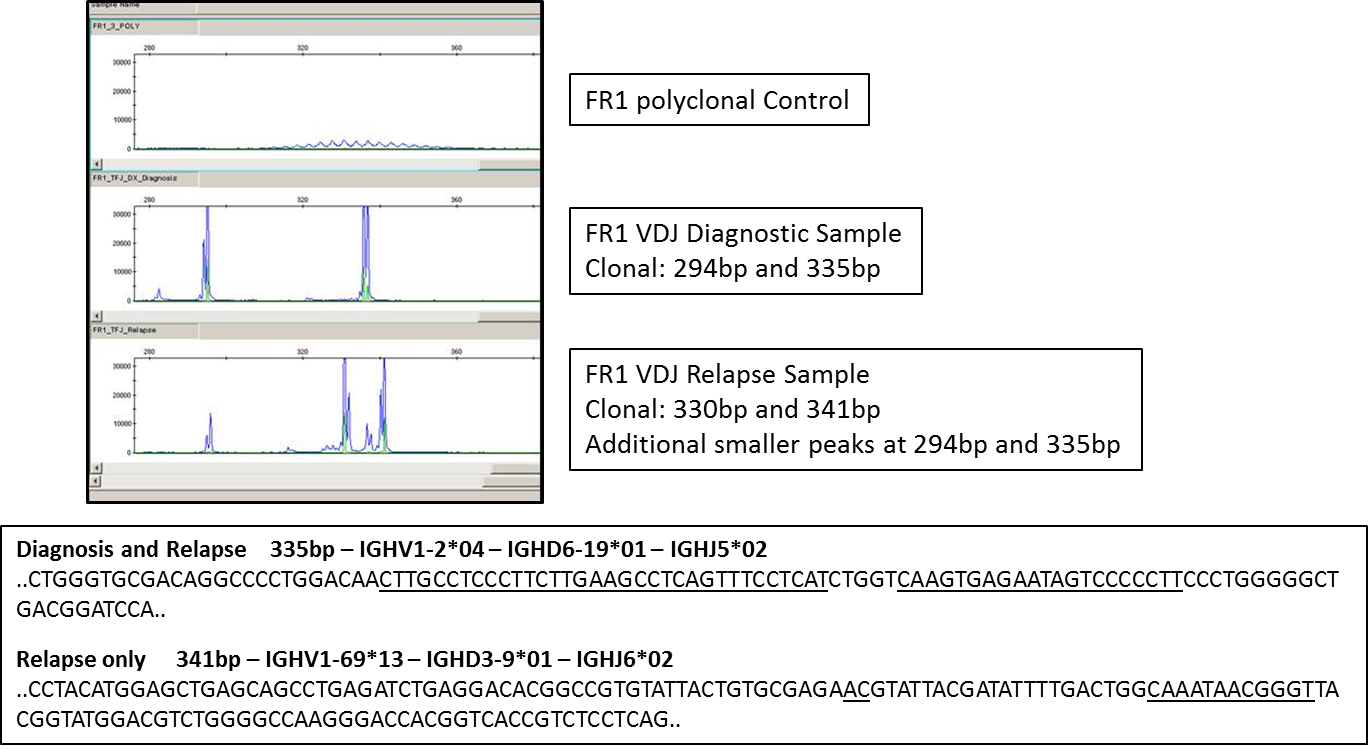


Ford et al Supplementary Information

**Figure S4 The *IGH* gene clonal rearrangement observed at relapse is not detectable by Q-PCR at diagnosis**

Sequence of 341bp *IGH* gene rearrangement only observed at relapse (see Figure S3) and Q-PCR primers used: *Forward, Probe, Reverse.*

Random insertions of N’s at the VDJ joins are underlined.

TACTGTGCGAGAACGTATTACGATATTTTGACTGGCAAATAACGGGTTACGGTATGGACGTCTGGGGCCAAGGGAC

Q-PCR plot from Bio-Rad CFX96.

**
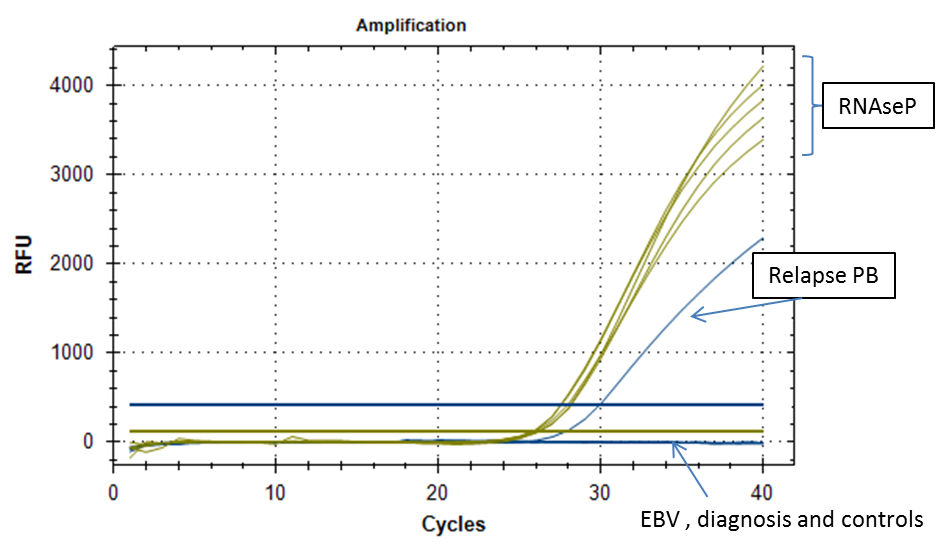
**

Ford et al Supplementary Information

**Figure S5 IGV browser screenshots for *IKZF1;***

The Figure shows ***IKZF1*** at diagnosis (dx, 50 reads, heterozygous deletion) versus relapse (50 reads, larger heterozygous deletion).


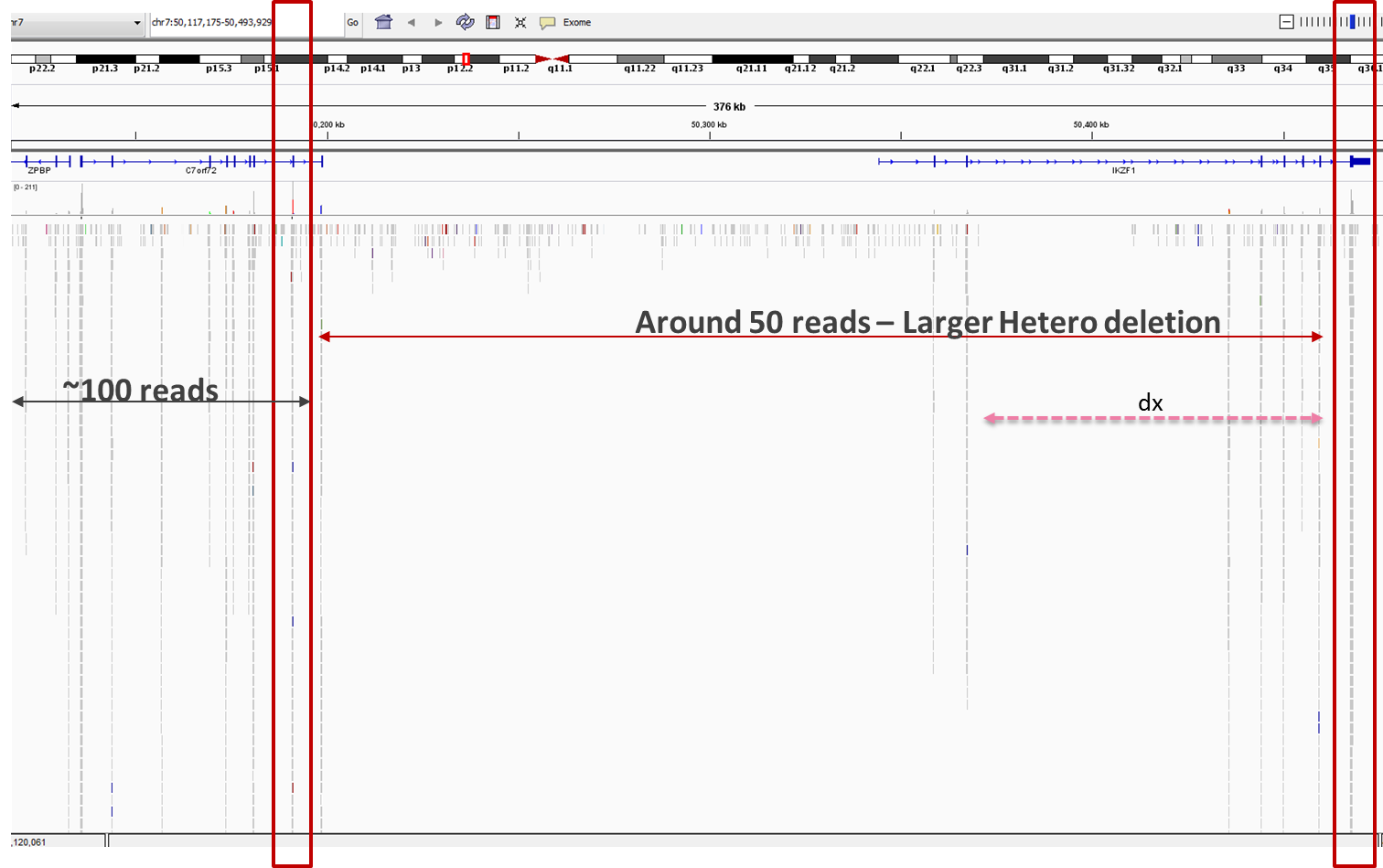

Supplement: Supplementary Information [file leu2015132x1.doc]
